# Cost analysis of single-use (Ambu^®^ aScope™) and reusable bronchoscopes in the ICU

**DOI:** 10.1186/s13613-016-0228-3

**Published:** 2017-01-03

**Authors:** S. Perbet, M. Blanquet, C. Mourgues, J. Delmas, S. Bertran, B. Longère, V. Boïko-Alaux, P. Chennell, J.-E. Bazin, J.-M. Constantin

**Affiliations:** 1 Réanimation Adultes & USC, Pôle de Médecine Péri-Opératoire, CHU Clermont-Ferrand, Clermont-Ferrand, France; 2 R2D2, EA 7281, Faculté de Médecine, Université d’Auvergne, Clermont-Ferrand, France; 3 Service de Santé Publique, CHU Clermont-Ferrand, 7, Place Henri-Dunant, 63058 Clermont-Ferrand Cedex 1, France; 4 Clermont Université, Université d’Auvergne, EA 4681, PEPRADE (Périnatalité, grossesse, Environnement, PRAtiques médicales et DEveloppement), Clermont-Ferrand, France; 5 Pharmacie centrale, Centrale d’Approvisionnement de Matériel Stérile, CHU Clermont-Ferrand, Clermont-Ferrand, France; 6 Réanimation Médico-Chirugicale, Pôle de Médecine Péri-Opératoire, Hôpital Gabriel-Montpied, CHU Clermont-Ferrand, 58 Rue Montalembert, 63000 Clermont-Ferrand Cedex, France

**Keywords:** Fiberscope, Medico-economic analysis, Single use, Percutaneous tracheostomy, Airway management

## Abstract

**Background:**

Flexible optical bronchoscopes are essential for management of airways in ICU, but the conventional reusable flexible scopes have three major drawbacks: high cost of repairs, need for decontamination, and possible transmission of infectious agents. The main objective of this study was to measure the cost of bronchoalveolar lavage (BAL) and percutaneous tracheostomy (PT) using reusable bronchoscopes and single-use bronchoscopes in an ICU of an university hospital. The secondary objective was to compare the satisfaction of healthcare professionals with reusable and single-use bronchoscopes.

**Methods:**

The study was performed between August 2009 and July 2014 in a 16-bed ICU. All BAL and PT procedures were performed by experienced healthcare professionals. Cost analysis was performed considering ICU and hospital organization. Healthcare professional satisfaction with single-use and reusable scopes was determined based on eight factors. Sensitivity analysis was performed by applying discount rates (0, 3, and 5%) and by simulation of six situations based on different assumptions.

**Results:**

At a discount rate of 3%, the costs per BAL for the two reusable scopes were 188.86€ (scope 1) and 185.94€ (scope 2), and the costs per PT for the reusable scope 1 and scope 2 and single-use scopes were 1613.84€, 410.24€, and 204.49€, respectively. The cost per procedure for the reusable scopes depended on the number of procedures performed, maintenance costs, and decontamination costs. Healthcare professionals were more satisfied with the third-generation single-use Ambu^®^ aScope™.

**Conclusions:**

The cost per procedure for the single-use scope was not superior to that for reusable scopes. The choice of single-use or reusable bronchoscopes in an ICU should consider the frequency of procedures and the number of bronchoscopes needed.

**Electronic supplementary material:**

The online version of this article (doi:10.1186/s13613-016-0228-3) contains supplementary material, which is available to authorized users.

## Background

Flexible optical bronchoscopes are essential for management of airways when performing bronchoalveolar lavage (BAL), intubation, airway suctioning, and percutaneous tracheostomy (PT) in intensive care units (ICUs) [1–3]. However, the conventional reusable flexible scopes have three major drawbacks: high cost of repairs, need for decontamination, and possible transmission of Creutzfeldt–Jakob disease or other infectious agents [4, 5]. The single-use flexible Ambu^®^ aScope™ (Ambu A/S, Ballerup, Denmark) is designed for tracheal intubation and has the advantages that repair and disinfection are not needed [6]. Nonetheless, research is needed to assess the efficacy of this new device and to determine that it meets the current standards for reusable flexible bronchoscopes. Piepho et al. [6] studied the aScope™ and reported lower success rate for intubation and limited capacity to perform airway suctioning. Two additional drawbacks of the aScope™ are availability of only one size and its low image resolution [7, 8]. Mankikian found a global satisfaction with another type of single-use devices in clinical practice and for BAL in ventilated piglets [9].

Three previous studies performed cost-effectiveness or cost-minimization analysis to compare reusable flexible scopes with single-use scopes. Gupta et al. [10] and Aïssou et al. [11] concluded that single-use scopes should be considered as alternative devices and that their cost range was similar to reusable scopes. Tvede et al. [12] reported that single-use scopes were more expensive than reusable scopes in their institution, but this depended on the number of flexible optic intubations per year; the cost for disposable scopes was lower if there were only few intubations per year. However, these studies had several limitations, in that they did not consider all costs or cost discounting and they did not perform sensitivity analyses.

The research team of our university hospital reviewed these previous studies and hypothesized it would be better to have both types of scopes available, rather than one or the other. They developed a protocol to test this hypothesis based on BAL and PT procedures that were performed in an ICU that considered the advantages and drawbacks of each scope.

The main objective of this study was to measure cost per BAL performed by reusable scopes and the cost per PT performed by reusable and single-use scopes in an ICU of a university hospital. The secondary objective was to measure healthcare professional satisfaction with the three generations of single-use scopes.

## Methods

### Setting

The study was performed in the ICU of a single institution (University Hospital of Clermont-Ferrand, Clermont-Ferrand, France) that had 16 beds. Two reusable fiberscopes, an Olympus^®^ LF-TP (Olympus Optical Co. Ltd, Tokyo, Japan) and a Pentax^®^ FI-16BS (Pentax France, Argenteuil), were available for all kinds of airways management. They were listed as fiberscopes 1 and 2 (Fib1 and Fib2). The single-use Ambu^®^ aScope™ was available for PT only. The study was performed between August 2009 and July 2014. All procedures (BAL and PT) were performed in the ICU by senior physicians.

Because this study was observational and did not change the daily practice, the Research Ethics Committee of the Hospital of Cannes approved the present study and waived informed consent was authorized (Research Ethics Committee of the Hospital of Cannes, ceth_s03). However, the patient’s next of kin was systematically orally informed and could refuse patient participation. Moreover, the patient was later and as soon as possible systematically informed and could refuse his data to be used.

### Materials

The two reusable scopes required decontamination after each use. There were two decontamination procedures, depending on the type of scope and the duration between uses, according to the national guidelines of the Health General Direction. Decontamination procedures were manual, because no mechanical washer disinfector was available in our hospital. If a scope was used in the previous 7 days, a two-part 60-min incomplete decontamination procedure was performed: 20 min before use (including preparation, one soak, and cleaning) and 40 min after use (cleaning, three soaks with cleaning, drying, and storage). In other cases, a complete procedure of 75 min (35 min before use and 40 min after use) was performed. In case of damage, the reusable scope was sent to the manufacturer and no other scope replaced it.

Three generations of single-use aScopes were used: The first one could be used for 30-min maximum, the second one for 8-h maximum (limitation by manufacturer), and the third one with no limitation. A short screening decontamination of 5 min was performed as recommended.

### Cost measurement

For the reusable flexible scopes, the cost of purchase, tax write-off (amortization), insurance policy, repairs, and decontamination procedures were included (Additional file 1: Table S1). The purchase cost included the specific cost for each fiberscope and the common costs. The specific cost for the fiberscope 2 included the cost paid for the device, battery, and cable; the specific cost for the fiberscope 1 included the cost paid for the device, light, and cable. The common costs included storage bags for the devices and the impermeability tester. Only the Olympus scope had an insurance policy, and this was established in 2014. The assumption was that one-tenth of the total cost of the reusable scope was written off. The repair costs were paid to the manufacturer. The decontamination cost included the time that nurses spent performing the cleaning and the cost of cleaning materials. There were no waste management costs because these were the manufacturer’s responsibility.

For the single-use scope, the cost of purchase, decontamination, and waste management were included. The purchase cost was paid to the manufacturer. The decontamination costs included the time that nurses spent performing the cleaning and the costs of associated materials. Single-use scopes have a risk of being infectious after use and must be burned. The cost of burning one single-use scope was based on the known cost of burning 1 tonne of waste.

### Sensitivity analysis

A sensitivity analysis that considered all the assumptions made in the cost analysis was used to assess the robustness of our results. First, results were discounted at three different rates (0, 3, and 5%) by considering that all costs occurred at the beginning of the year, as recommend by Drummond et al. [13]. Second, the variables, type of scope, repairs, insurance policy, and number of airway management procedures (BAL or PT) were assessed in seven different simulations (see below). The results of the simulations are expressed at a discount rate of 3%. The annual repair costs used in the simulations were calculated from the total costs in the 5 years of the study: 3424€ for the fiberscope 1 and 3149€ for the fiberscope 2.

The first simulation modeled the current situation in our ICU without considering the insurance policy for the fiberscope 1. The second simulation modeled the use of the reusable and disposable scopes with equal frequency, repairs for reusable scopes, and no insurance for the fiberscope 1. The third simulation was the same as the second simulation, except without repairs and with an insurance policy for the fiberscope 1. The fourth and fifth simulations were the same as the second simulation, except for the more frequent use of the reusable scope 1 in the fourth simulation (2/3 vs. 1/3) and the more frequent use of the reusable scope 2 in the fifth simulation (2/3 vs. 1/3). The sixth simulation assessed the impact of an increase in repair costs with all other factors the same. These six simulations were performed for 100 airway management procedures per year, based on the yearly average activity of our ICU. The seventh simulation considered the evolution of cost per airway management for the reusable and disposable scopes, assuming that one reusable fiberscope is needed for 50 procedures. A modelisation was performed based on the average activity per year.

### Measurement of physician satisfaction

The satisfaction of healthcare professionals with the single-use scopes relative to reusable scopes was measured by a numeric scale from 0 (less satisfied with single-use scope) to 100 (more satisfied with single-use scope). The satisfaction scores were measured separately for the three generations of single-use scopes. This scoring considered eight factors, including implementation, anatomic landmarks, device insertion, tracheal positioning, quality of picture, luminosity, kickstand and maneuverability, and global satisfaction. Drawbacks and advantages were noted for each specific scope.

## Results

There were 518 airway management procedures performed during the 5-year study period. A total of 457 procedures were performed with reusable scopes (155 with the fiberscope 1 [136 BAL and 19 PT] and 302 with the fiberscope 2 [245 BAL and 57 PT]), and 61 PT procedures were performed with single-use scopes (15 with first-generation scopes, 30 with second-generation scopes, and 16 with third-generation scopes).

Additional files 2 and 3: Tables S2 and S3 show the detailed costs for the reusable and single-use scopes. The major cost for the reusable scopes was the purchase (Additional file 2: Table S2). The costs per BAL at a discount rate of 3% for the reusable scopes 1 and 2 were 188.86€ and 185.94€, respectively (Additional file 3: Table S3). The costs per PT at a discount rate of 3% for the reusable scopes 1 and 2 and single-use scopes were 1613.84€, 410.24€, and 204.49€, respectively (Additional file 3: Table S3). For the two reusable scopes, decontamination and maintenance were the main expenditures when performing BAL, and maintenance and write-offs were the main expenditures when performing PT. These results remained robust when different discount rates were applied (Additional file 3: Table S3).

**Fig. 1:**
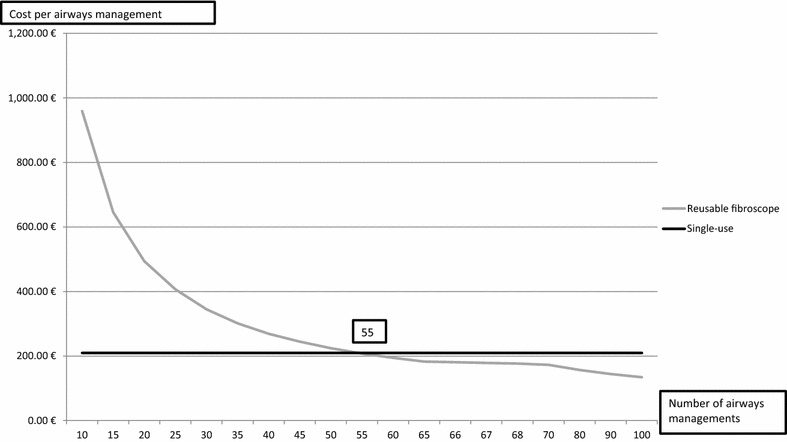
Evolution of cost per airways management (EUR) when using one reusable scope per 50 airways managements per year comparing with single-use scopes

The simulations indicated that the cost per year ranged from 15,668.82€ to 30,821.67€, depending on the assumptions (Additional file 4: Table S4). Simulation one showed that the cost per procedure for the scope 1 with an insurance policy was slightly lower than for the single-use scope. Simulations two, three, four, and five showed that as reusable scopes were used more often, the cost per procedure became close to or lower than that of the single-use scope. The cost per procedure remained robust when they were twice as the mean cost, for the scope 2 only (Simulation six in Additional file 4: Table S4). For reusable scopes, the cost per procedure dropped dramatically when the number of procedures increased except when the purchase of a second reusable device was needed (Fig. 1). The first break-even point was at 55 procedures (BAL + PT) per year, considering equivalent usage of the different reusable fiberscopes.

Healthcare professionals had overall satisfaction with the single-use Ambu^®^ aScope™ than compared with the reusable scopes (Table 1). Notably, the quality of the image, implementation, anatomic landmarks, device insertion, and tracheal positioning were correct, although luminosity, and kickstand and maneuverability were less satisfactory.

**Table 1:** Results of healthcare professionals’ satisfaction when using the three generations of Ambu^®^ aScope™ compared to reusable fiberscopes (value = 50)

	aScopeTM1			aScopeTM2			aScopeTM3		
	Median	IQR1	IQR3	Median	IQR1	IQR3	Median	IQR1	IQR3
Implementation	50	46.25	53.75	50	50	55	57.5	50	60
Anatomic landmarks	50	41.25	53.75	60	51.25	60	60	56.25	60
Device insertion	57.5	46.25	60	57.5	50	60	60	56.25	63.75
Tracheal positioning	55	50	60	60	51.25	60	60	51.25	60
Quality of picture	35	30	40	40	35	40	55	45	60
Luminosity	40	30	45	40	35	40	40	35	45
Kickstand and maneuverability	30	30	35	35	30	40	45	40	50
Global satisfaction	40	36.25	45	45	40	50	55	50	60

*IQR1* interquartile range 1, *IQR3* interquartile range 3

## Discussion

### Main results

Our analysis indicated that reusable scopes varied significantly in cost depending on the number of procedures performed per year and that cost for purchase and maintenance were most significant. The cost per procedure for the reusable and single-use scopes were comparable when there were 55 procedures per year. Our sensitivity analysis indicated that the cost for repairs can be reduced by an insurance policy, and this reduced expenses by about 900€ per year for the scope 1. A survey of healthcare professionals indicated greater satisfaction with the third-generation single-use scope than with the reusable scopes.

### Comparisons with other studies

Two previous studies reported that the cost per BAL for reusable scopes and the cost per PT for the single-use scope were comparable to our results. More specifically, Tvede et al. [12] measured a cost per intubation from 177.7€ to 204.5€ for reusable scopes and 204.4€ for disposable scopes. Aïssou et al. [11] calculated a cost per intubation for reusable and single-use scopes of 206€ and 200€, respectively. On the contrary, our costs were higher than reported by Gupta et al. [10], who measured the 2009 cost per intubation by a reusable scope as $119.75 (82.63€ based on the exchange rate of January 2009). The break-even point in the present work cannot be compared with that of Tvede et al. [12] due to the different settings. The same drop of decline of cost per airways management performed was observed for reusable scope also explaining by the higher capital expenditures represented by the purchase of these device.

A review of the literature indicated that previous studies examined the association between repair costs and number of procedures performed by experienced practitioners [14, 15]. The impact of an educational program on repair costs was also measured. Performance of such a program may help to reduce repair expenses for the reusable scopes.

### Implications

The cost per procedure for single-use scopes remained stable in our sensitivity analysis. The satisfaction of healthcare professionals was greater for the third-generation disposable scope. Two different sizes of disposable scopes are now available. The ICU in a teaching hospital has a mission to educate inexperienced practitioners, so single-use scopes may be a more suitable instrument for airway management.

### Strengths and limitations

The main strengths of our study are that we considered direct medical costs and that we performed sensitivity analysis to assess the robustness of the results. The main limitation is that we did not assess the effectiveness of the single-use scopes and that the choice of fibrescopes was appreciated by operators. A review of the literature indicates that further research is needed to assess the effectiveness of disposable scopes in daily practice [6–9, 16]. It is also important to measure bronchoscope failure rates and the medical consequences of failure on effectiveness and direct medical costs. We considered the limitation noted by Tvede et al. [12] in their measurement of the break-even point regarding the actual need for the device. However, we did not consider opportunity costs associated with decontamination by auxiliary nurses and automatic annual microbiological samples. Indeed, time spent for decontamination procedures can be reassigned to patient care. The costs were considered with manual decontamination procedures, but they could be determined equally in case of mechanical centralized decontamination procedures. Another limitation was that BAL was not performed with single-use fiberscope, because the two first generation have not operator channel and then use of them was not validated by the pharmacy for BAL.

## Conclusions

The cost per procedure for the single-use scope is comparable to that for reusable scopes. When an ICU is considering the use of reusable scopes or single-use scopes, it should consider the annual number of procedures and the number of scopes that are needed.

## Additional files

**Additional file 1.** Detailed cost of the decontamination procedures.

**Additional file 2.** Cost analysis for the reusable and the single-use scopes for bronchoalveolar lavage and tracheostomy in the intensive care unit during the six years of the study.

**Additional file 3.** Total cost and cost per bronchoalveolar lavage and tracheostomy for reusable and single-use scopes at discount rate of 0, 3, and 5%.

**Additional file 4.** Results of the sensitivity analysis at a discount rate of 3%.
