# Supplementary material for: Cost analysis of single-use (Ambu® aScope™) and reusable bronchoscopes in the ICU
Source: Ann Intensive Care. 2017 Jan 3;7:3. doi: 10.1186/s13613-016-0228-3 (PMC5209315; doi:10.1186/s13613-016-0228-3)
Supplement: Supplementary file 1 — Additional file 1. Detailed cost of the decontamination procedures. [file 13613_2016_228_MOESM1_ESM.docx]

**Table S1: Detailed cost of the decontamination procedures**

|  |  | **Reusable Pentax® scope** | | | |  | **Reusable Olympus® scope** | | | |
| --- | --- | --- | --- | --- | --- | --- | --- | --- | --- | --- |
|  |  | Incomplete  procedure | | Complete  procedure | |  | Incomplete  procedure | | Complete  procedure | |
|  | cost/unit | Unit | Total cost | Unit | Total cost | cost/unit | Unit | Total cost | Unit | Total cost |
| Sterile equipment | 0.43 | 2.00 | 0.86 | 2.00 | 0.86 |  | 2.00 | 0.86 | 2.00 | 0.86 |
| KIT 1-CLEAN® | 3.13 | 1.00 | 3.13 | 1.00 | 3.13 |  | 1.00 | 3.13 | 1.00 | 3.13 |
| Syringe 50cc BD PLASTIPAK® | 0.24 | 2.00 | 0.48 | 2.00 | 0.48 |  | 2.00 | 0.48 | 2.00 | 0.48 |
| Valve | 7.45 | 1.00 | 7.45 | 1.00 | 7.45 | 2.71 | 1.00 | 2.71 | 1.00 | 2.71 |
| Piston | 9.79 | 1.00 | 9.79 | 1.00 | 9.79 | - | - | - | - | - |
| Hand towel FOLIDRAPE® | 0.28 | 2.00 | 0.56 | 2.00 | 0.56 |  | 2.00 | 0.56 | 2.00 | 0.56 |
| Gloves | 0.17 | 4.00 | 0.68 | 4.00 | 0.68 |  | 4.00 | 0.68 | 4.00 | 0.68 |
| Sterile gloves | 0.30 | 2.00 | 0.60 | 2.00 | 0.60 |  | 2.00 | 0.60 | 2.00 | 0.60 |
| Protective cap and mask FFP1 | 0.32 | 1.00 | 0.32 | 1.00 | 0.32 |  | 1.00 | 0.32 | 1.00 | 0.32 |
| Antibacterial and fungicide Aniosurf premium® | 0.07 | 1.00 | 0.07 | 1.00 | 0.07 |  | 1.00 | 0.07 | 1.00 | 0.07 |
| Enzymatic decontamination product (1l) | 8.50 | 0.01 | 0.09 | 0.02 | 0.13 |  | 0.01 | 0.09 | 0.02 | 0.13 |
| Antibacterial and fungicide product (5l) | 19.80 | 0.50 | 9.90 | 0.50 | 9.90 |  | 0.50 | 9.90 | 0.50 | 9.90 |
| Sterile water 1 L | 0.47 | 9.00 | 4.23 | 9.00 | 4.23 |  | 9.00 | 4.23 | 9.00 | 4.23 |
| Auxiliary nurse (1 hour) | 21.64 | 1.00 | 21.64 | 1.25 | 27.05 |  | 1.00 | 21.64 | 1.25 | 27.05 |
| **Total** |  |  | **59.80** |  | **65.25** |  |  | **45.27** |  | **50.72** |
