# Supplementary material for: Cost analysis of single-use (Ambu® aScope™) and reusable bronchoscopes in the ICU
Source: Ann Intensive Care. 2017 Jan 3;7:3. doi: 10.1186/s13613-016-0228-3 (PMC5209315; doi:10.1186/s13613-016-0228-3)
Supplement: Supplementary file 2 — Additional file 2. Cost analysis for the reusable and the single-use scopes for bronchoalveolar lavage and tracheostomy in the intensive care unit during the six years of the study. [file 13613_2016_228_MOESM2_ESM.docx]

**Table S2: Cost analysis for the reusable and the single-use scopes for bronchoalveolar lavage and tracheostomy in the Intensive Care Unit during the six years of the study**

|  | **2009** | | | **2010** | | | **2011** | | | **2012** | | | **2013** | | | **2014** | | |
| --- | --- | --- | --- | --- | --- | --- | --- | --- | --- | --- | --- | --- | --- | --- | --- | --- | --- | --- |
|  | n^b^ | c/u^c^ | total | n^b^ | c/u^c^ | total | n^b^ | c/u^c^ | total | n^b^ | c/u^c^ | total | n^b^ | c/u^c^ | total | n^b^ | c/u^c^ | total |
| **BAL^a^** |  |  |  |  |  |  |  |  |  |  |  |  |  |  |  |  |  |  |
| **Reusable scope 1** |  |  |  |  |  |  |  |  |  |  |  |  |  |  |  |  |  |  |
| Purchase | 1 | 9095 | 9095 | 0 | 0.0 | 0.0 | 0 | 0.0 | 0.0 | 0 | 0.0 | 0.0 | 0 | 0.0 | 0.0 | 0 | 0.0 | 0.0 |
| Amortization | 1 | 909.5 | 909.5 | 1 | 909.5 | 909.5 | 1 | 909.5 | 909.5 | 1 | 909.5 | 909.5 | 1 | 909.5 | 909.5 | 1 | 909.5 | 909.5 |
| Insurance policy | 0 | 0.0 | 0.0 | 0 | 0.0 | 0.0 | 0 | 0.0 | 0.0 | 0 | 0.0 | 0.0 | 0 | 0.0 | 0.0 | 1 | 2500.0 | 2500.0 |
| Maintenance | 1 | 3600.0 | 3600.0 | 0 | 0.0 | 0.0 | 0 | 0.0 | 0.0 | 0 | 0.0 | 0.0 | 0 | 0.0 | 0.0 | 0 | 0.0 | 0.0 |
| Incomplete decontamination | 0 | 43.1 | 0.0 | 22 | 43.5 | 956.7 | 28 | 43.9 | 1229.9 | 8 | 44.4 | 355.0 | 18 | 44.8 | 806.7 | 17 | 45.3 | 769.6 |
| Complete decontamination | 0 | 48.2 | 0.0 | 10 | 48.7 | 487.2 | 13 | 49.2 | 639.8 | 4 | 49.7 | 198.8 | 8 | 50.2 | 401.7 | 8 | 50.7 | 405.8 |
| **Reusable scope 2** |  |  |  |  |  |  |  |  |  |  |  |  |  |  |  |  |  |  |
| Purchase | 1 | 10780 | 10780 | 0 | 0.0 | 0.0 | 0 | 0.0 | 0.0 | 0 | 0.0 | 0.0 | 0 | 0.0 | 0.0 | 0 | 0.0 | 0.0 |
| Amortization | 1 | 1078.0 | 1078.0 | 1 | 1078.0 | 1078.0 | 1 | 1078.0 | 1078.0 | 1 | 1078.0 | 1078.0 | 1 | 1078.0 | 1078.0 | 1 | 1078.0 | 1078.0 |
| Maintenance | 0 | 0.0 | 0.0 | 1 | 3100.0 | 3100.0 | 1 | 7144.0 | 7144.0 | 1 | 450.0 | 450.0 | 1 | 6000.0 | 6000.0 | 0 | 0.0 | 0.0 |
| Incomplete decontamination | 12 | 56.9 | 682.4 | 47 | 57.4 | 2699.9 | 27 | 58.0 | 1566.6 | 52 | 58.6 | 3047.7 | 63 | 59.2 | 3729.7 | 27 | 59.8 | 1614.6 |
| Complete decontamination | 1 | 62.1 | 62.1 | 3 | 62.7 | 188.0 | 2 | 63.3 | 126.6 | 4 | 64.0 | 255.8 | 5 | 64.6 | 323.0 | 2 | 65.3 | 130.5 |
| **Tracheostomy** |  |  |  |  |  |  |  |  |  |  |  |  |  |  |  |  |  |  |
| **Reusable scope 1** |  |  |  |  |  |  |  |  |  |  |  |  |  |  |  |  |  |  |
| Purchase | 1 | 9095 | 9095 | 0 | 0.0 | 0.0 | 0 | 0.0 | 0.0 | 0 | 0.0 | 0.0 | 0 | 0.0 | 0.0 | 0 | 0.0 | 0.0 |
| Amortization | 1 | 909.5 | 909.5 | 1 | 909.5 | 909.5 | 1 | 909.5 | 909.5 | 1 | 909.5 | 909.5 | 1 | 909.5 | 909.5 | 1 | 909.5 | 909.5 |
| Insurance policy | 0 | 0.0 | 0.0 | 0 | 0.0 | 0.0 | 0 | 0.0 | 0.0 | 0 | 0.0 | 0.0 | 0 | 0.0 | 0.0 | 1 | 2500.0 | 2500.0 |
| Maintenance | 1 | 3600.0 | 3600.0 | 0 | 0.0 | 0.0 | 1 | 3800.0 | 3800.0 | 1 | 4500.0 | 4500.0 | 1 | 2500.0 | 2500.0 | 0 | 0.0 | 0.0 |
| Incomplete decontamination | 0 | 43.1 | 0.0 | 3 | 43.5 | 130.5 | 6 | 43.9 | 263.6 | 2 | 44.4 | 88.7 | 0 | 44.8 | 0.0 | 1 | 45.3 | 45.3 |
| Complete decontamination | 0 | 48.2 | 0.0 | 2 | 48.7 | 97.4 | 3 | 49.2 | 147.6 | 1 | 49.7 | 49.7 | 0 | 50.2 | 0.0 | 1 | 50.7 | 50.7 |
| **Reusable scope 2** |  |  |  |  |  |  |  |  |  |  |  |  |  |  |  |  |  |  |
| Purchase | 1 | 10780 | 10780 | 0 | 0.0 | 0.0 | 0 | 0.0 | 0.0 | 0 | 0.0 | 0.0 | 0 | 0.0 | 0.0 | 0 | 0.0 | 0.0 |
| Amortization | 1 | 1078.0 | 1078.0 | 1 | 1078.0 | 1078.0 | 1 | 1078.0 | 1078.0 | 1 | 1078.0 | 1078.0 | 1 | 1078.0 | 1078.0 | 1 | 1078.0 | 1078.0 |
| Maintenance | 0 | 0.0 | 0.0 | 0 | 0.0 | 0.0 | 1 | 600.0 | 600.0 | 0 | 0.0 | 0.0 | 1 | 3200.0 | 3200.0 | 0 | 0.0 | 0.0 |
| Incomplete decontamination | 20 | 56.9 | 1137.4 | 18 | 57.4 | 1034.0 | 4 | 58.0 | 232.1 | 7 | 58.6 | 410.3 | 5 | 59.2 | 296.0 | 0 | 59.8 | 0.0 |
| Complete decontamination | 1 | 62.1 | 62.1 | 1 | 62.7 | 62.7 | 0 | 63.3 | 0.0 | 1 | 64.0 | 64.0 | 0 | 64.6 | 0.0 | 0 | 65.3 | 0.0 |
| **Single-use flexible scope** |  |  |  |  |  |  |  |  |  |  |  |  |  |  |  |  |  |  |
| Purchase | 0 | 216.8 | 0.0 | 3 | 219.0 | 657.0 | 8 | 221.2 | 1769.8 | 13 | 223.5 | 2905.0 | 20 | 225.7 | 4514.4 | 17 | 228.0 | 3876.0 |
| Screen decontamination | 0 | 2.7 | 0.0 | 3 | 2.7 | 8.1 | 8 | 2.7 | 21.7 | 13 | 2.7 | 35.7 | 20 | 2.8 | 55.4 | 17 | 2.8 | 47.6 |
| Waste management | 0 | 0.1 | 0.0 | 3 | 0.1 | 0.2 | 8 | 0.1 | 0.5 | 13 | 0.1 | 0.8 | 20 | 0.1 | 1.2 | 17 | 0.1 | 1.0 |

*^a^* *bronchoalveolar lavage;* *b: number of unit; c cost per unit*
