# Supplementary material for: Cost analysis of single-use (Ambu® aScope™) and reusable bronchoscopes in the ICU
Source: Ann Intensive Care. 2017 Jan 3;7:3. doi: 10.1186/s13613-016-0228-3 (PMC5209315; doi:10.1186/s13613-016-0228-3)
Supplement: Supplementary file 3 — Additional file 3. Total cost and cost per bronchoalveolar lavage and tracheostomy for reusable and single-use scopes at discount rate of 0, 3, and 5%. [file 13613_2016_228_MOESM3_ESM.docx]

**Table S3: Total cost and cost per bronchoalveolar lavage and tracheostomy for reusable and single-use scopes at discount rate of 0%, 3% and 5%.**

|  |  | **TOTAL 6 years BAL** | | | | | |  | **TOTAL 6 years Tracheostomy** | | | | | |
| --- | --- | --- | --- | --- | --- | --- | --- | --- | --- | --- | --- | --- | --- | --- |
|  |  | **0%** | | **3%** | | **5%** | |  | **0%** | | **3%** | | **5%** | |
|  | **n^a^** | **cost** | **c/n^b^** | **cost** | **c/n** | **cost** | **c/n** | **n** | **cost** | **c/n** | **cost** | **c/n** | **cost** | **c/n** |
| **Reusable scope 1** | 136 | 26903.16 | **197.82** | 25684.81 | **188.86** | 24965.46 | **183.57** | 19 | 32325.53 | **1701.34** | 30665.84 | **1613.84** | 29676.53 | **1561.92** |
| Purchase |  | 9095.00 | 66.88 | 9095.00 | 66.88 | 9095.00 | 66.88 |  | 9095.00 | 478.68 | 9095.00 | 478.68 | 9095.00 | 478.68 |
| Writing off |  | 5457.00 | 40.13 | 5074.74 | 37.31 | 4847.16 | 35.64 |  | 5457.00 | 287.21 | 5074.74 | 267.09 | 4847.16 | 255.11 |
| Insurance policy (2014) |  | 2500.00 | 18.38 | 2156.52 | 15.86 | 1958.82 | 14.40 |  | 2500.00 | 131.58 | 2156.52 | 113.50 | 1958.82 | 103.10 |
| Maintenance (2009-2013) |  | 3600.00 | 26.47 | 3600.00 | 26.47 | 3600.00 | 26.47 |  | 14400.00 | 757.89 | 13521.22 | 711.64 | 12990.74 | 683.72 |
| Total decontamination |  | 6251.16 | 45.96 | 5758.54 | 42.34 | 5464.49 | 40.18 |  | 873.53 | 45.98 | 818.35 | 43.07 | 784.82 | 41.31 |
| **Reusable scope 2** | 245 | 48368.98 | **197.42** | 45554.92 | **185.94** | 43868.23 | **179.05** | 57 | 24346.43 | **427.13** | 23383.55 | **410.24** | 22809.62 | **400.17** |
| Purchase |  | 10780.00 | 44.00 | 10780.00 | 44.00 | 10780.00 | 44.00 |  | 10780.00 | 189.12 | 10780.00 | 189.12 | 10780.00 | 189.12 |
| Writing off |  | 6468.00 | 26.40 | 6014.92 | 24.55 | 5745.18 | 23.45 |  | 6468.00 | 113.47 | 6014.92 | 105.52 | 5745.18 | 100.79 |
| Maintenance |  | 16694.00 | 68.14 | 15486.35 | 63.21 | 14757.14 | 60.23 |  | 3800.00 | 66.67 | 3408.72 | 59.80 | 3176.87 | 55.73 |
| Total decontamination |  | 14426.98 | 58.89 | 13273.64 | 54.18 | 12585.91 | 51.37 |  | 3298.43 | 57.87 | 3179.91 | 55.79 | 3107.57 | 54.52 |
| **Single-use scope** |  | - |  | - |  | - |  | 61 | 13894.42 | **227.78** | 12473.63 | **204.49** | 11635.61 | **190.75** |
| Purchase |  | - |  | - |  | - |  |  | 13722.29 | 224.96 | 12319.10 | 201.95 | 11491.46 | 188.38 |
| Screen decontamination |  | - |  | - |  | - |  |  | 168.52 | 2.76 | 151.29 | 2.48 | 141.12 | 2.31 |
| Waste management |  | - |  | - |  | - |  |  | 3.61 | 0.06 | 3.24 | 0.05 | 3.02 | 0.05 |

*^a^n: number of procedures, ^b^c/n: cost/number of procedures*
