# Supplementary material for: Cost analysis of single-use (Ambu® aScope™) and reusable bronchoscopes in the ICU
Source: Ann Intensive Care. 2017 Jan 3;7:3. doi: 10.1186/s13613-016-0228-3 (PMC5209315; doi:10.1186/s13613-016-0228-3)
Supplement: Supplementary file 4 — Additional file 4. Results of the sensitivity analysis at a discount rate of 3%. [file 13613_2016_228_MOESM4_ESM.docx]

**Table S4: Results of the sensitivity analysis at a discount rate of 3% (Fib1= fiberscope 1, Fib2= fiberscope 2)**

| **Simulation 1:** Repairs Fib2: Yes + Insurance Fib 1: Yes | | | | | | |
| --- | --- | --- | --- | --- | --- | --- |
| **ICU current airways management distribution for 100 procedures (n)** | | | **Cost/year (euros)** | **Cost/airways management (euros)** | | |
| Fib 1 | Fib 2 | Single-use |  | Fib 1 | Fib 2 | Single-use |
| 30 | 58 | 12 | 17737.26 | 202.18 | 157.88 | 209.56 |
| **Simulation 2:** Repairs Fib 1 and Fib 2: Yes + Insurance Fib 1: No | | | | | | |
| **Airways management distribution for 100 procedures 50%Fib1//50% Fib2 (n)** | | | **Cost/year (euros)** | **Cost/airways management (euros)** | | |
| Fib 1 | Fib 2 | Single-use |  | Fib 1 | Fib 2 | Single-use |
| 0 | 0 | 100 | 20955.54 | 0 | 0 | 209.56 |
| 10 | 10 | 80 | 29088.73 | 600.54 | 631.89 | 209.56 |
| 20 | 20 | 60 | 25948.57 | 323.25 | 345.51 | 209.56 |
| 30 | 30 | 40 | 22808.41 | 230.82 | 250.05 | 209.56 |
| 35 | 35 | 30 | 21238.33 | 204.41 | 222.78 | 209.56 |
| 40 | 40 | 20 | 19668.25 | 184.61 | 202.32 | 209.56 |
| 45 | 45 | 10 | 18098.17 | 169.20 | 186.41 | 209.56 |
| 50 | 50 | 0 | 16528.09 | 156.88 | 173.68 | 0 |
| **Simulation 3:** Repairs Fib 2: Yes + insurance Fib 1: Yes | | | | | | |
| **Airways management distribution for 100 procedures 50%Fib1//50% Fib2 (n)** | | | **Cost/year (euros)** | **Cost/airways management (euros)** | | |
| Fib 1 | Fib 2 | Single-use |  | Fib 1 | Fib 2 | Single-use |
| 0 | 0 | 100 | 20955.54 | 0 | 0 | 209.56 |
| 10 | 10 | 80 | 28229.46 | 514.61 | 631.89 | 209.56 |
| 20 | 20 | 60 | 25089.30 | 280.29 | 345.51 | 209.56 |
| 30 | 30 | 40 | 22094.29 | 202.18 | 250.05 | 209.56 |
| 35 | 35 | 30 | 20379.06 | 179.86 | 222.78 | 209.56 |
| 40 | 40 | 20 | 18808.98 | 163.13 | 202.32 | 209.56 |
| 45 | 45 | 10 | 17238.90 | 150.11 | 186.41 | 209.56 |
| 50 | 50 | 0 | 15668.82 | 139.69 | 173.68 | 0 |
| **Simulation 4:** Repairs Fib1 and Fib 2: Yes + insurance Fib 1: No | | | | | | |
| **Airways management distribution for 100 procedures 1/3Fib1//2/3Fib2** | | | **Cost/year (euros)** | **Cost/airways management (euros)** | | |
| Fib 1 | Fib 2 | Single-use |  | Olympus | Fib 2 | Single-use |
| 0 | 0 | 100 | 20955.54 | 0 | 0 | 209.56 |
| 5 | 15 | 80 | 29154.57 | 1155.12 | 440.97 | 209.56 |
| 10 | 30 | 60 | 26080.25 | 600.54 | 250.05 | 209.56 |
| 20 | 40 | 40 | 22940.09 | 323.25 | 202.32 | 209.56 |
| 25 | 45 | 30 | 21370.01 | 267.79 | 186.41 | 209.56 |
| 25 | 55 | 20 | 19865.76 | 267.79 | 163.27 | 209.56 |
| 30 | 60 | 10 | 18295.68 | 230.82 | 154.59 | 209.56 |
| 30 | 70 | 0 | 16791.44 | 230.82 | 140.95 | 0 |
| **Simulation 5:** Repairs Fib1 and Fib 2: Yes + Insurance Fib 1: No | | | | | | |
| **Airways management distribution for 100 procedures 2/3Fib1//1/3Fib2** | | | **Cost/year (euros)** | **Cost/airways management (euros)** | | |
| Fib1 | Fib 2 | Single-use |  | Fib1 | Fib 2 | Single-use |
| 0 | 0 | 100 | 20955.54 | 0 | 0 | 209.56 |
| 15 | 5 | 80 | 29022.89 | 415.68 | 1204.65 | 209.56 |
| 30 | 10 | 60 | 25816.90 | 230.82 | 631.89 | 209.56 |
| 40 | 20 | 40 | 22676.74 | 184.61 | 345.51 | 209.56 |
| 45 | 25 | 30 | 21106.66 | 169.20 | 288.23 | 209.56 |
| 55 | 25 | 20 | 19470.74 | 146.80 | 288.23 | 209.56 |
| 60 | 30 | 10 | 17900.66 | 138.39 | 250.05 | 209.56 |
| 70 | 30 | 0 | 16264.74 | 125.19 | 250.05 | 0 |
| **Simulation 6:** Repairs Fib1 and Fib2: Yes, Mean cost*1, Mean cost*2, Mean cost*3 | | | | | | |
| **Repairs mean cost** | **ICU current airways management distribution** | | **Cost/year (euros)** | **Cost/airways management (euros)** | | |
|  | Fib1/Fib 2/single-use | |  | Fib1 | Fib 2 | Single-use |
| Mean cost | 30/58/12  30/58/12  30/58/12 | | 18596.53 | 230.82 | 157.88 | 209.56 |
| Mean cost*2 |  |  | 24709.10 | 336.96 | 208.37 | 209.56 |
| Mean cost*3 |  |  | 30821.67 | 443.10 | 258.86 | 209.56 |

*ICU: intensive care unit*

*Simulation 1 kept the ICU current airways management distribution, reparation for the fiberscope 2 and insurance for the fiberscope 1. In these conditions,*

*the cost per year was 17737.26€. Costs per airway management performed were for fiberscope 1, fiberscope 2 and single-use, 202.18 €, 157.88€ and 209.56€, respectively. Simulation 2 chose an airways management distribution of 50%/50% for the fiberscopes 1 and 2, reparation for the both fiberscope and no insurance for the fiberscope 1. When the fiberscopes 1 and 2 were used for 35 airways management procedures, the cost per year was 21238.33€ and costs per airways management were for fiberscope 1, fiberscope 2 and single-use, 204.41€, 222.78€ and 209.56€, respectively. Simulation 3 chose an airways management distribution of 50%/50% for the fiberscopes 1 and 2, reparation for the fiberscope 2 and insurance for the fiberscope 1. When the fiberscopes 1 and 2 were used for 35 airways management procedures, the cost per year was 20379.06€ and costs per airways management were for fiberscope 1, fiberscope 2 and single-use, 179.86€, 222.78€ and 209.56€, respectively. Simulation 4 chose an airways management distribution of 1/3Fiberscope1//2/3Fiberscope 2, reparation for the both fiberscopes and no insurance for fiberscope 1. When the fiberscopes 1 was used for 20 management procedures, the fiberscope 2 for 40 airways management procedures and the single-use fiberscope for 40 airways management procedures the cost per year was 22940.09€ and costs per airways management were for fiberscope 1, fiberscope 2 and single-use, 323.25€, 202.32€ and 209.56€, respectively. Simulation 5 chose an airways management distribution of 2/3Fiberscope1//1/3Fiberscope 2, reparation for the both fiberscopes and no insurance for fiberscope 1. When the fiberscopes 1 was used for 45 management procedures, the fiberscope 2 for 25 airways management procedures and the single-use fiberscope for 30 airways management procedures, the cost per year was 21106.66€ and costs per airways management were for fiberscope 1, fiberscope 2 and single-use, 169.20€, 288.23€ and 209.56€, respectively. Simulation 6 chose the ICU current airways management distribution and increased repair mean cost for the both fiberscopes were evaluated. In this last simulation, when the repair mean cost doubled, the cost per year increase from 18596.53 euros to 24709.10 euros and costs per airways management procedure increased for fiberscope 1 and fiberscope 2 from 230.82 euros and 157.88 euros to 336.96 euros and 208.37 euros, respectively. The cost of single-use fiberscope remained the same all along the different simulations.*
